# Long-Term Efficacy of Combined Focused and Radial Extracorporeal Shockwave Therapy for Gluteus Medius Tendon Pathology: A Pilot Study

**DOI:** 10.3390/life14121698

**Published:** 2024-12-21

**Authors:** Federica Fulceri, Larisa Ryskalin, Gabriele Morucci, Francesco Busoni, Paola Soldani, Marco Gesi

**Affiliations:** 1 Department of Translational Research and New Technologies in Medicine and Surgery, University of Pisa, Via Roma 55, 56126 Pisa, Italy; federica.fulceri@unipi.it (F.F.); larisa.ryskalin@unipi.it (L.R.); gabriele.morucci@unipi.it (G.M.); paola.soldani@unipi.it (P.S.); 2 Studio Radiologico Busoni, Private Practice, 56125 Pisa, Italy; f.busoni@yahoo.it

**Keywords:** combined shockwave therapy, ultrasonography, gluteal tendinopathy, radial shockwave, focused shockwave, functional outcomes, sports medicine

## Abstract

**Background:** Gluteus medius tendinopathy is amongst the most prevalent lower limb tendinopathies and is now recognized as the primary cause of insidious lateral hip pain. Typically affecting middle-aged women, this condition causes disability and reduced quality of life as it negatively impacts most daily life activities. Several studies demonstrate that extracorporeal shockwave therapy is effective in reducing pain and promoting functional recovery in several musculoskeletal disorders including tendinopathies. However, most published data are limited to evaluating focal or radial shockwaves as single interventions. Contrariwise, there is little evidence reporting the use of combined ESWT treatment and outcomes for managing tendon pathologies, and no data are reported on combined ESWT for gluteus medius tendinopathy. **Objectives:** The aim of this study was to evaluate the clinical outcomes of combined ESWT in gluteus medius tendinopathy. **Methods:** Medical charts of 11 consecutive patients with gluteal tendinopathy confirmed by ultrasound who underwent a combined ESWT protocol were reviewed. Changes in pain severity and lower limb function were evaluated using the numerical rating scale, the Victorian Institute of Sports Assessment for Gluteal tendinopathy questionnaire, and the Roles and Maudsley score. Clinical outcome measurements were collected at baseline (T0), 2 months after combined ESWT (T1), and at long-term follow-up (T2), at least 10 months post-treatment (mean 26 months). **Results:** The mean age of the sample was 62.55 ± 3.17 years. A marked prevalence of females was recorded (nine subjects, 81.8%). A significant improvement was observed in all outcome criteria both at short- and long-term follow-up after combined ESWT compared to baseline (*p* < 0.05). Treatment success rates were 90.9% and 81.8% at T1 and T2, respectively. **Conclusions:** Combined ESWT is effective and safe for patients with gluteal tendinopathy, with good long-term results in terms of pain relief and improved functional impairment.

## 1. Introduction

Degenerative changes, including tears, of the tendon insertions of hip abductors, including gluteus medius and minimus muscles, are recognized as the primary cause of symptoms in patients suffering from greater trochanteric pain syndrome [1,2,3]. In particular, gluteus medius (Gmed) tendon pathology is among the most prevalent of all lower limb tendinopathies [4,5,6], with a prevalence ranging from 10 to 25% of the general population [7,8]. Of note, a recent study performed in Dutch general medical practice found gluteal tendinopathy to have the highest prevalence (4.22 per 1000 person years) and incidence (3.29 per 1000 person years) of all presenting lower limb tendinopathies [9]. In particular, gluteal tendinopathy represents the most common cause of lateral-sided hip pain and tenderness of insidious onset, with an important impact on patients’ participation in daily living activities [9,10,11,12]. Although Gmed tendinopathy typically affects mid-life sedentary individuals, elite athletes and sports practitioners (particularly runners) might also be affected [11,13]. In particular, this condition predominantly arises in middle-aged women, in their fourth to sixth decades of life, regardless of their activity level [2,3,14]. Patients affected by Gmed tendinopathy usually report exacerbated symptoms during daily life activities such as lying on the affected side or common weight-bearing tasks, making it a debilitating musculoskeletal condition [3,6]. Remarkably, Gmed tendinopathy can be challenging to manage if clinically misdiagnosed as other soft tissue pathologies around the greater trochanter may mimic lateral hip pain [11,14,15,16]. This, in turn, may result in delayed or inappropriate therapeutic intervention for this clinical condition and erroneous conclusions regarding treatment effectiveness. On the other hand, radiological investigations to detect structural abnormalities at the proximal gluteus medius enthesis on the posterior iliac crest are clinically difficult due to the complex anatomy of the hip region and overlapping spectrum of symptoms [15]. Thus, accurate diagnosis and the precise identification of the localization and extent of pain are crucial to facilitate the most appropriate and successful rehabilitation protocol.

In recent decades, a large body of literature has demonstrated that extracorporeal shockwave therapy (ESWT) can induce various molecular and biological effects on bone and soft tissues through mechano-transduction [17,18,19,20,21]. For instance, increasing evidence indicates that ESWT has a beneficial effect on tissue repair and regeneration by regulating stem cell activities as well as protein synthesis, cell proliferation, differentiation, and migration within bones and soft tissues [21,22,23]. ESWT can also promote neovascularization by increasing the expression of growth factors related to angiogenesis such as vascular endothelial growth factor and nitric oxide, thus improving blood flow and local tissue perfusion [24,25,26]. Furthermore, ESWT can modulate the immune response and cellular inflammation by regulating macrophage activity, leukocyte infiltration, cytokine, and chemokine production [26,27,28]. Again, there are several clinical observational studies on the beneficial effects of ESWT on pain relief in different acute and chronic musculoskeletal pain conditions [18,22,25].

Thus, ESWT has become a popular noninvasive and safe therapeutic intervention to reduce pain and promote functional recovery in several musculoskeletal disorders, such as lateral epicondylitis, calcific and non-calcific shoulder tendinitis, Achilles tendinopathy, and plantar fasciitis [22,29,30,31,32,33,34,35,36]. In general, two different types of ESWT are used in clinical practice, namely, radial and focal shockwave therapy, which differ in terms of the device used, the characteristics of the generated waveform, the depth of impact, and energy intensities [28]. While most research reports the use of focal or radial ESWT in isolation as single treatment modalities, a few recent studies demonstrate promising results in terms of improvement in pain and function when both devices are used in combination within the very same treatment session (i.e., combined shockwave therapy) [37,38,39,40,41].

Notwithstanding the growing popularity of shockwave therapy as a conservative intervention for several lower limb tendinopathies, clinical evidence regarding the long-term effectiveness of ESWT in treating gluteal tendinopathy is still limited [16,42,43]. At the same time, published data aim to evaluate radial or focal shockwave therapy delivered as single interventions [42,43,44,45], while to the best of our knowledge, no previous studies have reported the use of combined treatment for Gmed tendinopathy patients.

Therefore, the objective of the present retrospective study was to describe the functional outcomes and safety of combined ESWT in Gmed tendinopathy. The outcomes were documented by ultrasonographic appearance of the gluteus medius tendon, as well as subjective pain and functional scores.

## 2. Materials and Methods

### 2.1. Subjects and Clinical Assessment

This retrospective study included all patients diagnosed and treated with ESWT for Gmed tendinopathy at the Center for Rehabilitative Medicine “Sport and Anatomy” of the University of Pisa between September 2020 and December 2023. Patient characteristics, treatment measures, and functional outcomes were extracted by 3 authors (F.F., L.R., and M.G.) using chart review in all patients receiving combined ESWT.

Regarding symptoms, patients reported pain with an insidious onset, that manifests chronically, intermittently, or continuously, at the proximal lateral aspect of the hip, which may even radiate to the distal thigh. Pain and tenderness on palpation of the greater trochanter were regarded as the main diagnostic criteria for gluteal tendinopathy [13].

Clinical assessment of Gmed tendinopathy was confirmed by ultrasonography examination by a radiologist (F.B.) with over 20 years of experience in musculoskeletal ultrasound. Diagnostic evaluations were performed using a high-resolution ultrasound machine (Mindray DC-70 EXP, Mindary, Shenzhen, China). Gluteus medius tendon pathology was determined when the US showed one or more of the following abnormal findings: decreased and heterogeneous echogenicity of the gluteus medius and/or gluteus minimus tendons, tendon thickening, occurrence of enthesophytes, bone erosions, or soft tissue calcifications. Both proximal and distal gluteus medius insertions were documented and included in the present study. The exclusion criteria were a previous history of fracture or acute trauma in the affected limb, hip or spinal surgery, comorbidities (e.g., neurological, vascular, or rheumatological disorders), age <18 years, and general contraindications to shockwave therapy [46]. Exclusion criteria also included the inability to have outcome measures available. The study was conducted in accordance with local legislation and institutional requirements, and informed consent was obtained from all participants.

### 2.2. ESWT Protocol

The patients underwent a cycle of combined ESWT using a Duolith SD1 (Storz Medical AG., Tägerwilen, Switzerland). Three to five sessions were administered at weekly intervals. ESWT was performed with the patient lying on the unaffected side, with the affected thigh slightly flexed. ESWT was applied at the relevant (patient-oriented focusing) location, which corresponded topographically with the gluteal (outer) surface of the ilium and/or the lateral aspect of the greater trochanter. In detail, combined treatment consisted of a sequential application of f-SWT and r-SWT for each therapeutic session. f-SWT was performed targeting primarily the gluteus medius entheses and tendon by delivering 1000 shocks at 5 Hz with an energy flux density (EFD) of 0.20 mJ/mm^2^, while r-SWT targeted the myotendinous junction of the affected muscle and the fascia of the lateral-posterior thigh district by delivering 2000 shocks at 14 Hz with an EFD of 1.8 (range 1.6–2.0) mJ/mm^2^. An ultrasound transmission gel was placed between the shockwave applicator and the skin. No local anesthesia was applied according to the ISMST procedure [47]. All the patients were able to complete the treatment with no reported side effects. Patients were instructed to avoid the use of NSAIDs during the treatment. Patients were also discouraged from performing pain-provoking, heavy activities during the entire cycle of treatment, and required to slowly return to previous activity levels forty days after the last treatment session.

### 2.3. Outcome Measures

Clinical outcome measurements were collected at baseline (T0), at short-term follow-up 2 months (T1) after ESWT, and at long-term follow-up (T2), at least 10 months posttreatment (26.0 ± 9.5 months, mean ± SD). The primary outcome measure included the change in pain severity measured on an 11-point numerical rating scale (NRS-p; grading scale: 0 = no pain; 10 = worst conceivable pain).

The secondary outcomes included the following:

(1) Roles and Maudsley score (R&M score), a treatment satisfaction scale widely used when reporting results of ESWT. In detail, self-perceived overall pain and activity limitation with the R&M score was recorded with a 4-point system where “1 = excellent result”, “2 = good, significant improvements”, “3 = fair, somewhat improved”, and “4 = poor, symptoms identical or worse than before treatment” [30]. The success rate was calculated at each follow-up. Both good and excellent grades in RMS were considered as treatment success [43,48].

(2) Lower limb function assessment using the Victorian Institute of Sports Assessment for Gluteal tendinopathy (VISA-G) questionnaire, which represents a reliable and valid tool that specifically measures the severity of gluteal tendinopathy-related disability [49,50]. VISA-G consists of eight items, covering the domains of pain, function, and current activity. Scores range from 0 to 100 points, with higher scores being desirable as indicating less pain and better function. Thus, a score approaching 100 points represents a fully functional asymptomatic individual.

### 2.4. Statistical Analysis

An a priori power analysis was performed to determine the required sample size using G*Power software (version 3.1.9.6). The a priori sample size calculation computed a sample size of n = 4 for power of 95% (power 1-β err prob = 0.8; effect size = 2.24).

The Kruskal–Wallis test (non-parametric analysis of variance) was performed to compare the differences between the initial and follow-up measurements after combined ESWT. Linear regression analysis was used to evaluate correlations between patients’ age and symptom severity at T0, T1, and T2. Spearman’s correlation coefficient (ρ) was used to determine the degree of association. The linear correlation was performed using Microsoft Excel (version 16.56). Statistical analyses were performed with SPSS Statistics version 29.0.2.0 (IBM, New York, NY, USA). The null hypothesis (H0) was rejected when the *p*-value was less than 0.05. Data were reported as the mean ± SEM.

## 3. Results

**Figure 1:**
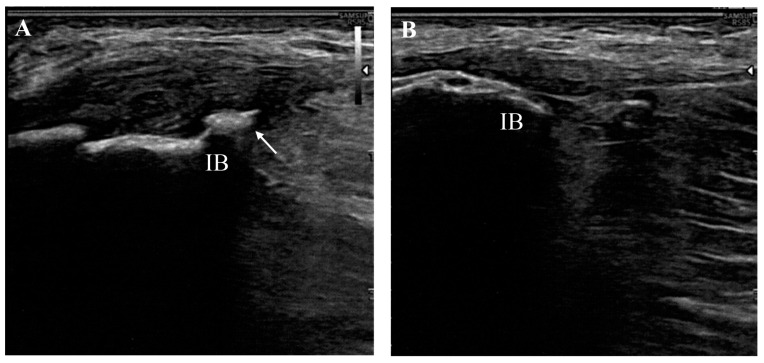
Long-axis image of a tendinopathic gluteus medius tendon (**A**) with contralateral healthy comparison (**B**) showing hypoechoic thickening at the attachment onto the iliac bone (IB) and irregularities of the bone margin due to the presence of an enthesophyte (arrow).

A chart review from two providers identified 14 consecutive patients (12 females and 2 males; mean age 64.36 ± 3.28 years) with ultrasound-confirmed gluteal tendinopathy who were treated with combined ESWT during the study period (Figure 1).

Of the 14 eligible patients, 3 patients were excluded because they could not be reached during the follow-up. Eventually, 11 subjects were included in the final analysis (Table 1 and Table 2).

**Figure 2:**
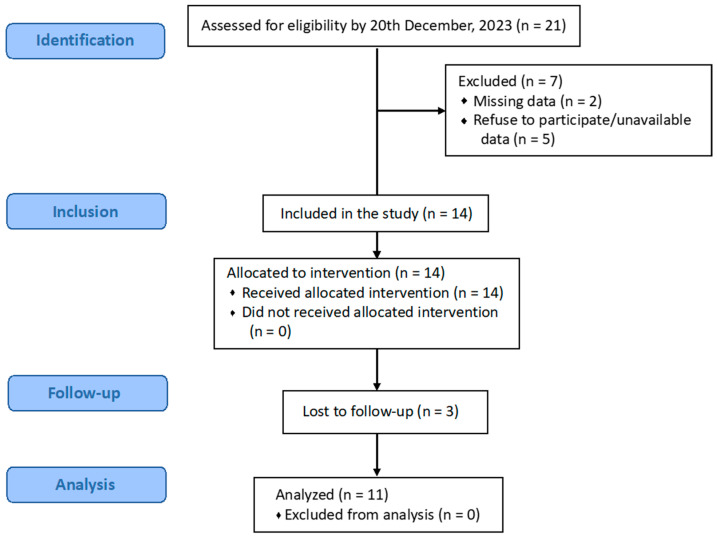
Flow diagram of participant recruitment, allocation, and analysis.

Figure 2 shows the flow diagram of this study based on the STROBE guidelines.

**Figure 3:**
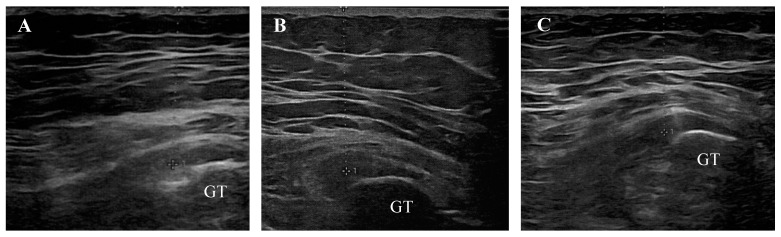
Transverse ultrasound views of pathological attachments of gluteus medius tendons onto the greater trochanter (GT). Representative images of tendinosis (**A**), enthesopathy (**B**), and enthesopathy accompanied by tendinosis (**C**).

The descriptive data and clinical diagnosis according to the ultrasound examination of the sample are summarized in Table 3 and Figure 3.

At baseline, the NRS-p score was 6.7 ± 0.6, the VISA-G score was 41.2 ± 6.4, and the R&M score was 3.4 ± 0.2, as reported in Figure 3, which overall indicates severe pain and disability. No statistically significant differences between genders were observed at T0 (Table 4).

**Figure 4:**
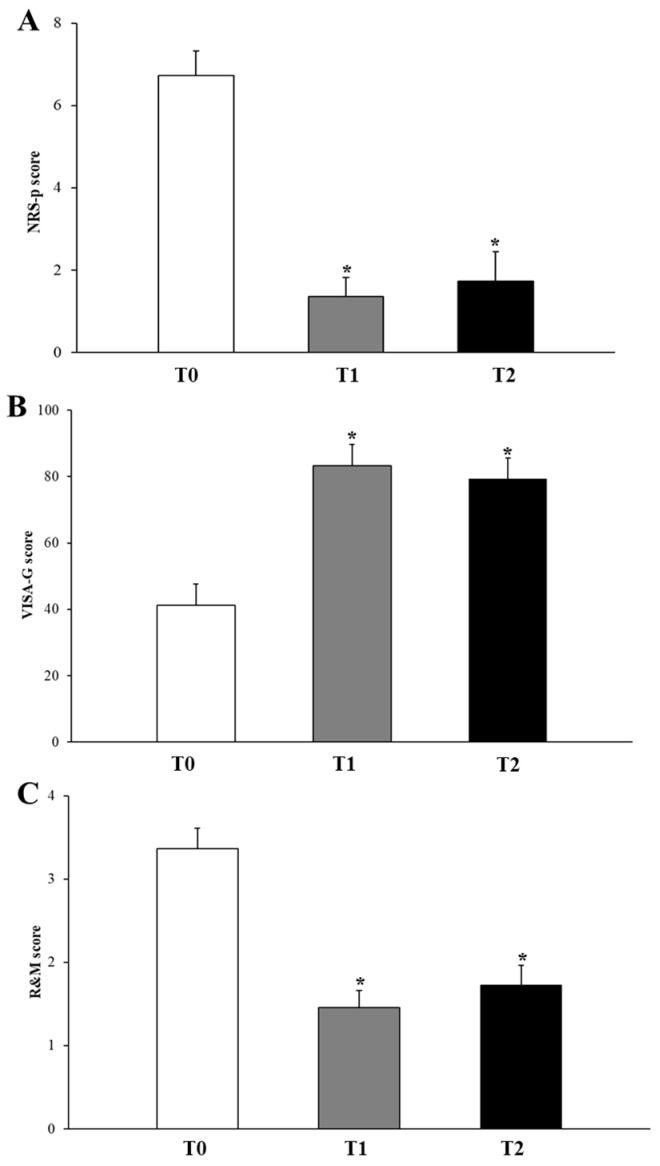
Average clinical outcome variations following c-SWT across time points of the study. NRS-p score (**A**), VISA-G (**B**), and R&M score (**C**). * *p* < 0.05 vs. T0.

Compared to the initial score, significant improvements were detected in all the aspects considered in the evaluation two months after ESWT treatment (T1). In detail, a significant decrease in pain intensity occurred at T1 when the NRS-p score dropped significantly down to 1.4 ± 0.4 (*p* < 0.001) (Figure 4A). At the same time, marked recovery in hip discomfort was observed as the VISA-G score significantly increased up to 83.3 ± 6.4 (*p* < 0.001), indicating substantial amelioration in quality of life and activities of daily living (Figure 4B). In line with this, the R&M score improved at T1 to 1.4 ± 0.2 (*p* < 0.001), suggesting a patient’s positive response regarding the effectiveness of ESWT treatment at short-term follow-up (Figure 4C). All these effects of ESWT treatment were preserved throughout the whole period of the study. In fact, no significant differences (*p* > 0.05) between short- and long-term follow-up were reported in NRS-p (1.7 ± 0.7; *p* = 0.9), VISA-G (79.4 ± 6.2; *p* = 0.6), and R&M scores (1.7 ± 0.2; *p* = 0.5) (Figure 4).

**Figure 5:**
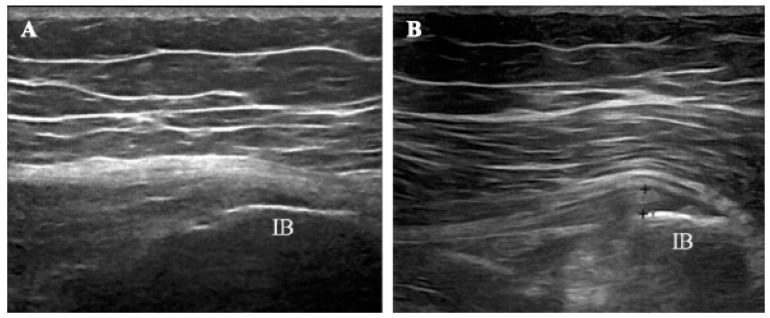
Longitudinal views of the gluteus medius tendon from a patient in the study before (**A**) and after (**B**) combined ESWT. At baseline, the ultrasound image showed hypoechoic thickening at the tendon’s insertion onto the iliac bone (IB). Irregularities of the tendon’s superior contour were also evident. In contrast, a marked improvement in the morphological aspect of the tendon was evident at T2, as shown by the more homogeneous tendon fibrillar pattern and reduced thickness.

Treatment success rates were 90.9% and 81.8% at short- (T1) and long-term (T2) follow-up, respectively. Ultrasonography revealed a trend of reduction in hypoechoic thickening and an increase in fibrillar echotexture in gluteus medius tendons after combined ESWT (Figure 5). Furthermore, no serious adverse events were recorded, and combined ESWT was well tolerated by all patients.

**Figure 6:**
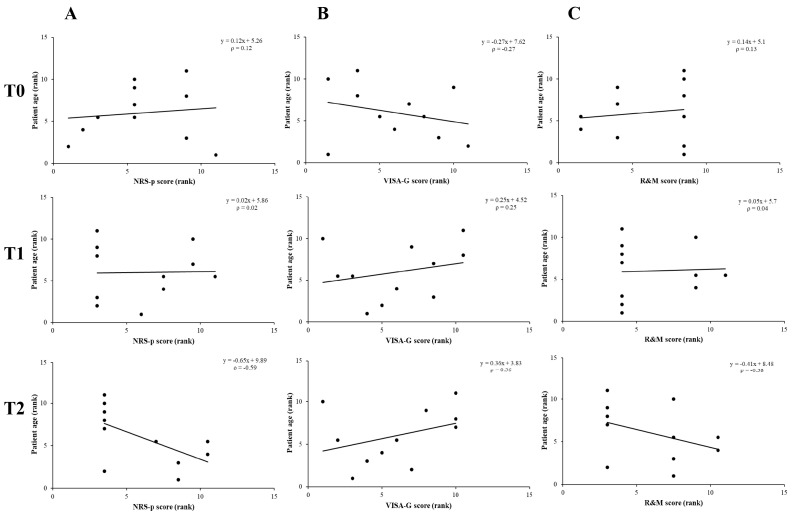
Linear correlation charts between outcome measures and patients’ age at different follow-ups following combined ESWT. NRS-p score (**A**), VISA-G score (**B**), and R&M score (**C**).

Lastly, the results of the Spearman rho test showed no significant correlation between age and outcome measures (Figure 6); however, a tendency of improvement in pain and functional outcomes with increasing age was noticed at long-term follow-up (T2).

## 4. Discussion

Currently, gluteus medius (Gmed) tendinopathy is among the most prevalent lower limb tendinopathies and represents a frequent cause of insidious lateral hip pain and tenderness on palpation along the greater trochanter. Patients with Gmed tendinopathy typically report difficulty lying on their side at night, standing/sitting, walking, and climbing stairs. This, in turn, may result in a severe disability that negatively impacts the overall quality of life. Among the recommended first-line treatments, ESWT has gained increasing attention in recent decades as a safe and effective conservative alternative in the therapeutic management of tendinopathies [12]. Within this frame, both radial and focused shockwave therapy were applied for the treatment of Gmed tendinopathy. However, evidence to support the use of ESWT in this musculoskeletal condition is extremely limited [16,42,43,44,45]. Regarding the therapeutic efficacy of r-SWT in Gmed tendinopathy treatment, Rompe et al. [42] found that low-energy ESWT (2000 pulses; 0.12 mJ/mm^2^ per session; three weekly sessions) led to significantly better results in pain relief than corticosteroid injections or home training exercises at short-term follow-up examination, with a beneficial effect that increased over months and reaching a success rate of 74% at 15 months. Similarly, Furia et al. [16] showed that a single low-energy treatment (2000 pulses; 0.18 mJ/mm^2^) was more effective in reducing pain and disability in the mid- (3 months) and long-term (12 months) perspectives compared to a control group managed with traditional nonoperative measures.

According to the screened existing literature, we could find only three studies regarding the clinical efficacy of focused ESWT in patients affected by GTPS. In particular, a randomized clinical trial (RCT) showed that patients with GTPS treated with piezoelectric focused shockwaves (1800 pulses; 0.15 mJ/mm^2^; 4 Hz; three weekly sessions) had better outcomes for pain relief at 2 and 6 months than those treated with ultrasound therapy [44]. However, with respect to lower limb functional improvement, focused ESWT did not prove to be superior to ultrasound therapy as no statistical differences were found in the comparison between groups [44]. An RCT by Ramon et al. [45] showed that three sessions of electromagnetic focused ESWT (2000 pulses; 0.20 mJ/mm^2^) are effective in improving pain, functional, and quality of life scores, with a success rate of 86.8% at 2 months after treatment, which was maintained throughout the follow-up period of 6 months. Again, the therapeutic efficacy of electrohydraulic low-energy focused ESWT in MRI-documented Gmed tendinopathy was assessed in a recent retrospective study by Seo et al. [43]. Contrary to former studies, the authors applied a treatment protocol that consisted of reducing the total energy per each session (60 mJ/mm^2^, much less than in previous studies) while increasing the number of treatment sessions, up to a maximum of 12. Although the positive effects achieved by the authors in the short term were comparable with those of former reports, they reported a tendency in subjective pain to increase with time, resulting in a slight drop in terms of treatment success between immediate and long-term follow-up (83.3% vs. 55.6%, respectively).

As reported in a recent systematic review by Ladurner et al. [2], to date, there is still a paucity of good clinical studies in this field of research, and no standard protocol has been yet established to provide the optimal ESWT treatment for this lower limb tendinopathy. At the same time, so far, there is no evidence for the use of combined focused and radial shockwave therapy for this pathological condition. As reported by previous studies, these latter devices act in somewhat different ways, as the focused ESWT can penetrate deeper into the tissue, whereas the radial ESWT dissipates radially at the skin and thus being more superficial [18]. However, it cannot be ruled out that the combined method may be effective as the two different forms of shockwaves act on different anatomical structures for a given musculoskeletal condition [46,51]. In particular, focused ESWT can generate interstitial and extracellular responses producing several beneficial effects on the tendon, including analgesia, tissue regeneration, neovascularization, and improvement of blood flow which further stimulate the regenerative process of the tissue [22]. For instance, focused ESWT was found to increase collagen turnover and accelerate tissue healing [52,53]. In addition, focused ESWT has a stimulating effect on the expression of lubricin in tendon sheath and septa, which in turn may contribute to the beneficial effects of shockwave in providing pain and symptom relief in musculoskeletal disorders by decreasing erosive wear [54]. On the other hand, radial ESWT was shown to increase muscular microcirculation, angiogenesis, and lymphatic drainage in a way that is similar to certain manual therapy techniques [24]. Positive effects were also observed in pain reduction and increased function in different chronic tendon injuries after exposure to radial ESWT [55,56]. At the same time, radial ESWT is effective in alleviating immobilization-induced contracture and fibrosis of muscle, as well as reducing the molecular manifestations of muscle fibrosis [57]. Thus, it can be assumed that the use of focused and radial shockwave therapy in combination within the very same treatment session may result in cumulative positive therapeutic effects on tissue regeneration, pain relief, and improved functional outcomes in injured tissues.

Within this frame, a few recent reports have found the dual treatment method feasible and effective for tendinopathy and fasciopathy [38,40,41,46]. For instance, in a prospective study, Saxena et al. [38] showed that combination therapy is a safe and improved method of treatment for Achilles tendinopathy compared to the isolated use of focused and radial shockwave therapy. Similarly, Robinson et al. [40] suggested that combined ESWT may provide more predictable functional gains for treatment of Achilles tendinopathy compared to radial ESWT. Combined therapy emerged as the best therapy in calcific shoulder tendinopathy compared to interventions alone [41]. Contrariwise, DeLuca et al. [39] reported similar functional gains using either combined or radial ESWT in the management of plantar fasciitis. Our findings are consistent with the existing literature reporting improvements with shockwave therapy for patients with Gmed tendinopathy. In particular, our study showed that combined ESWT results in early pain reduction, the improvement of functional and quality of life scores, and treatment satisfaction from the short-term (2 months) perspectives, with satisfactory improvements that were maintained for at least 1 year post-treatment. However, to our knowledge, the present retrospective study is the first to evaluate the long-term functional outcomes and safety of the combination shockwave therapy for the management of patients with Gmed tendinopathy documented by ultrasound, as former reports only report the use of single treatment modalities.

Nonetheless, some limitations in our study should be considered when interpreting the present findings: first, the small sample size; second, the lack of a control or placebo group. Further studies involving large-scale RTCs are needed to verify whether patients affected by Gmed tendinopathy who received the dual treatment method may experience more favorable outcomes in terms of pain relief and lower limb functional gains compared with the isolated use of focal or radial shockwave therapy. The third limitation is the lack of ultrasonography measurements at baseline and follow-ups. Further studies to correlate the clinical improvements with objective imaging techniques would broaden the understanding of the morpho-functional improvements obtained by shockwave therapy across various musculoskeletal disorders.

## 5. Conclusions

Keeping in mind the aforementioned limitations, this study provides valuable data to guide clinical practice, showing that patients with Gmed tendinopathy who received combined shockwave therapy experience pain relief and improvement in functional outcomes at long-term perspectives. Further studies are needed to underlie the molecular mechanisms through which combined shockwave therapy promotes the beneficial effects on tendon healing, which is still a challenging field of research.

## Figures and Tables

**Table 1:** Baseline characteristics of the entire sample. Values are shown as mean ± SEM (range) when relevant.

Variable	Mean ± SEM (Range)
Sex: female (n), %	9; 81.8%
Age (years)	62.5 ± 3.2 (32–73)
Weight (kg)	63.2 ± 3.7 (51.0–78.0)
Height (m)	1.7 ± 0.02 (1.58–1.85)
BMI (kg/m^2^)	22.4 ± 1.01 (19.37–25.40)

**Table 2:** Baseline characteristics of the patients based on gender. Values are shown as mean ± SEM (range) when relevant.

Variable	Mean ± SEM	Mean ± SEM
Sex	Female	Male
n (%)	9 (81.8%)	2 (18.2%)
Age (years)	61.7 ± 4.5	67.0 ± 0.0
Weight (kg)	60.1 ± 3.7	77.5 ± 0.5
Height (m)	1.65 ± 0.03	1.8 ± 0.01
BMI (kg/m^2^)	22.0 ± 1.23	24.0 ± 0.3

**Table 3:** Descriptive data and clinical characteristics of the entire sample.

Patient Number	Age	Gender	Duration of Symptoms	Pathological Insertion Site	Pathology
1	58	F	≤3 months	Distal	Enthesopathy + Tendinosis
2	49	F	3–6 months	Proximal	Enthesopathy + Tendinosis
3	67	M	≤3 months	Distal	Tendinosis
4	67	M	≤3 months	Distal	Tendinosis
5	71	F	3–6 months	Distal	Tendinosis
6	69	F	≤3 months	Proximal	Tendinosis
7	32	F	≤3 months	Distal	Enthesopathy
8	68	F	3–6 months	Distal	Enthesopathy
9	73	F	≤3 months	Proximal and distal	Enthesopathy
10	72	F	≥6 months	Distal	Enthesopathy
11	62	F	≥6 months	Distal	Enthesopathy

**Table 4:** Characteristics of the patients based on gender at T0. Values are shown as mean ± SEM.

Variable	Female	Male	*p*-Value
NRS-p score	6.9 ± 0.7	6.0 ± 1.0	0.6
VISA-G score	40.0 ± 7.6	46.5 ± 9.5	0.7
R&M score	3.4 ± 0.2	3.0 ± 1.0	0.5

## Data Availability

The data supporting the conclusions of the current study are available from the corresponding author upon reasonable request.
